# Oncogenic KRAS^G12D^ Transfer from Platelet-like Particles Enhances Proliferation and Survival in Non-Small Cell Lung Cancer Cells

**DOI:** 10.3390/ijms26073264

**Published:** 2025-04-01

**Authors:** Jorge Ceron-Hernandez, Gonzalo Martinez-Navajas, Jose Manuel Sanchez-Manas, María Pilar Molina, Jiajun Xie, Inés Aznar-Peralta, Abel Garcia-Diaz, Sonia Perales, Carolina Torres, Maria J. Serrano, Pedro J. Real

**Affiliations:** 1 Gene Regulation, Stem Cells and Development Group, GENyO, Pfizer-University of Granada-Andalusian Regional Government Centre for Genomics and Oncological Research, Avenida de la Ilustración 114, 18016 Granada, Spain; jorge.ceron@genyo.es (J.C.-H.); gm24@sanger.ac.uk (G.M.-N.); jose.sanchez@genyo.es (J.M.S.-M.); jiajun.xie@genyo.es (J.X.); sopero@ugr.es (S.P.); ctp@ugr.es (C.T.); 2 Liquid Biopsies and Cancer Interception Group, PTS, Granada GENyO, Pfizer-University of Granada-Andalusian Regional Government Centre for Genomics and Oncological Research, Avenida de la Ilustración 114, 18016 Granada, Spain; maria.molina@genyo.es (M.P.M.); ines.aznar@genyo.es (I.A.-P.); abel.garcia@genyo.es (A.G.-D.); 3 Department of Biochemistry and Molecular Biology I, Faculty of Science, University of Granada, Avenida Fuentenueva s/n, 18071 Granada, Spain; 4 Instituto de Investigación Biosanitaria ibs.GRANADA, 18012 Granada, Spain; 5 Department of Biochemistry and Molecular Biology III and Immunology, Faculty of Medicine, University of Granada, Avenida de la Investigación 11, 18016 Granada, Spain; 6 Molecular Pathology Lab. Intercenter Anatomical Pathology Unit, San Cecilio and Virgen de las Nieves University Hospitals, 18016 Granada, Spain

**Keywords:** KRAS, platelet-like-particles, non-small cell lung cancer, oncogene transfer

## Abstract

In the tumor context, platelets play a significant role in primary tumor progression, dissemination and metastasis. Analysis of this interaction in various cancers, such as non-small cell lung cancer (NSCLC), demonstrate that platelets can both transfer and receive biomolecules (e.g. RNA and proteins) to and from the tumor at different stages, becoming tumor-educated platelets. To investigate how platelets are able to transfer oncogenic material, we developed in vitro platelet-like particles (PLPs), from a differentiated MEG-01 cell line, that stably carry RNA and protein of the KRAS^G12D^ oncogene in fusion with GFP. We co-cultured these PLPs with NSCLC H1975 tumor cells to assess their ability to transfer this material. We observed that the generated platelets were capable of stably expressing the oncogene and transferring both its RNA and protein forms to tumor cells using qPCR and imaging techniques. Additionally, we found that coculturing PLPs loaded with GFP-KRAS^G12D^ with tumor cells increased their proliferative capacity at specific PLP concentrations. In conclusion, our study successfully engineered an MEG-01 cell line to produce PLPs carrying oncogenic GFP-KRAS^G12D^ simulating the tumor microenvironment, demonstrating the efficient transfer of this oncogene to tumor cells and its significant impact on enhancing proliferation.

## 1. Introduction

The intricate interplay between platelets and tumor cells has emerged as a critical factor in cancer development, progression and metastasis. While platelets are traditionally known for their role in hemostasis [1,2], they are increasingly recognized for their multifaceted contributions to tumor biology [3,4]. Platelets can act as carriers of tumor-derived proteins, nucleic acids and extracellular vesicles, potentially influencing the tumor microenvironment and accelerating cancer progression [5,6,7].

Recently, increasing attention has been given to the potential role of platelets in transferring both their own cargo and oncogenic material to tumor cells [8,9]. This platelet-mediated transfer of oncogenic cargo may contribute to tumor heterogeneity and impacts responses to therapy [10,11,12,13]. Consequently, platelets can promote tumor cell survival, proliferation and metastasis through multiple mechanisms, including protecting cancer cells from immune surveillance and facilitating their dissemination in the bloodstream [14,15].

Non-small cell lung cancer (NSCLC), which accounts for approximately 85% of all lung cancer cases, remains the leading cause of cancer-related deaths worldwide [16]. Despite significant advances in targeted therapies and immunotherapies, the prognosis for patients with advanced NSCLC remains poor, with a 5-year survival rate of less than 20% [17]. The molecular complexity of NSCLC, characterized by diverse genomic alterations, presents both challenges and opportunities for therapeutic intervention [18,19].

*KRAS* mutations are among the most frequent oncogenic drivers in NSCLC, present in 25–30% of cases [20]. These mutations have been shown to drive tumor cell proliferation, survival and metastasis through the activation of various downstream signaling pathways [21,22]. In particular, the “KRAS G12D” variant (*KRAS^G12D^*) is highly prevalent and is linked to poor prognosis and resistance to targeted therapies [23]. Additionally, studies utilizing platelets as liquid biopsy have demonstrated that mutated *KRAS* variants originating from various tumor types can be transferred to platelets [5,6].

Recent advances in understanding the genomic landscape of NSCLC and other cancer types have revealed the complexity of tumor evolution and the development of resistance mechanisms [23,24,25]. The concept of tumor heterogeneity, both spatial and temporal, plays a critical role in shaping treatment strategies and influencing patient outcomes [26]. In this context, the potential contribution of platelets to intra-tumoral heterogeneity through the transfer of oncogenic material represents a compelling area of research [9,27]. This interaction between platelets and tumor cells could be particularly relevant in the lungs, where recent studies have demonstrated that platelets are produced in significant quantities through immature megakaryocytes residing in the lung, especially in inflammation contexts [28].

In this study, we demonstrate the role of oncogenic *KRAS^G12D^* loaded in platelet-like particles (PLPs) generated in vitro and its transfer to tumor cells, focusing on its impact on NSCLC progression and therapy resistance. We engineered the human megakaryoblastic cell line MEG-01 to express the GFP-KRAS^G12D^ fusion protein and examined the production of PLPs carrying this oncogene. Additionally, we investigated the transfer of GFP-KRAS^G12D^ from PLPs to H1975 lung adenocarcinoma cells and assessed the functional consequences in terms of cell proliferation.

By investigating the mechanisms of platelet-mediated oncogene transfer, our research provides new insights into platelet impact in NSCLC, potentially leading to novel therapeutic strategies.

## 2. Results

### 2.1. Generation and Phenotypic Characterization of MEG-01 Cell Models Undifferentiated and Differentiated and Their PLPs

**Figure 1:**
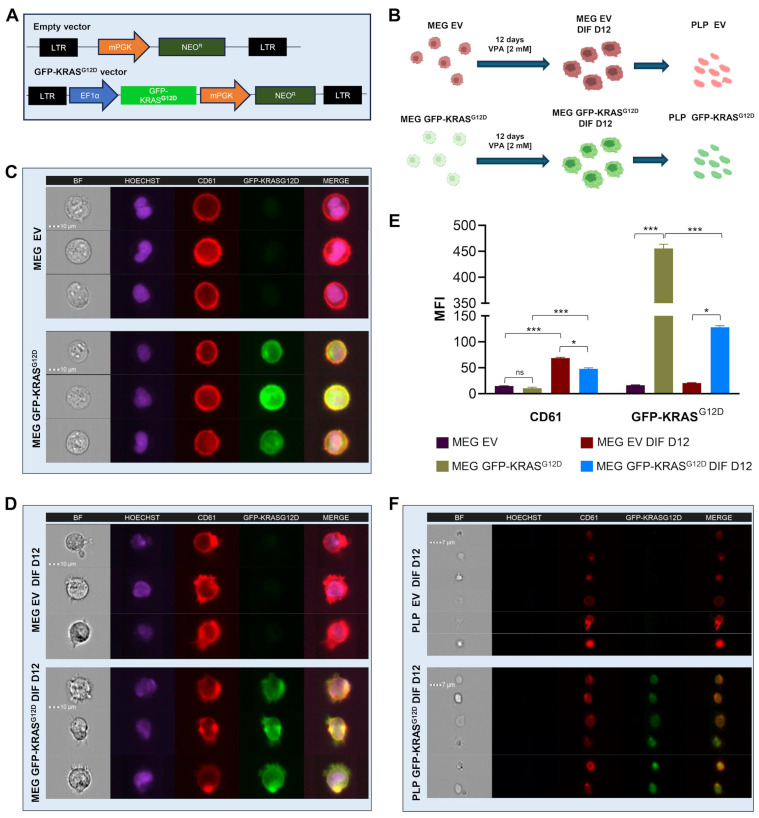
Generation and differentiation of MEG-01 cell line expressing the fusion protein GFP-KRAS^G12D^. (**A**) Schematic representation of the lentiviral vectors designs: Empty vector and GFP-KRAS^G12D^ vector. LTR: long terminal repeats. EF1α and mPGK: promoters. (**B**) Schematic representation of MEG-01 cell line differentiation and PLP production on day 12. (**C**) Representative ImageStream images of undifferentiated MEG-01 EV (top) and GFP-KRAS^G12D^ (bottom) cell lines. Channels indicate the localization and expression levels for Hoechst (nucleic DNA, purple), CD61 (red) and GFP-KRAS^G12D^ (green); the last channel corresponds to the merged channels, except for the brightfield. (**D**) Representative ImageStream images of differentiated MEG-01 EV (top) and GFP-KRAS^G12D^ (bottom) cell lines. Channels indicate the same as in panel (**C**). (**E**) Mean fluorescence intensity (MFI) comparison of CD61 (left) and GFP-KRAS^G12D^ (right) in MEG-01 EV and GFP-KRAS^G12D^ non-differentiated and differentiated. Data represent the mean ± SD for three independent experiments. Statistics were assessed with ANOVA two-way plus Tukey multiple comparison test (statistically significant differences: ns = non-significant, * *p* < 0.05, *** *p* < 0.001). (**F**) Representative ImageStream images of PLP EV (top) and GFP-KRAS^G12D^ (bottom) produced from MEG-01 on day 12. Channels indicate the same as in panel (**C**).

To investigate the potential role of platelets in transferring oncogenic KRAS^G12D^ to tumor cells, we constructed a lentiviral vector containing GFP and KRAS^G12D^ cDNAs fused in-frame, linking them with an HA-tag linker, along with a neomycin resistance cassette. We transduced the MEG-01 cell line to express either this GFP-KRAS^G12D^-expressing vector or the EV as a control (Figure 1A). The differentiation process of MEG-01 cells and subsequent production of PLPs were monitored over a 12-day period (Figure 1B).

Using the ImageStream platform, we were able to assess protein expression and localization at a single-cell level. This analysis confirmed the successful expression of GFP-KRAS^G12D^ in MEG-01-transduced cells. Representative images showed the localization and expression levels of Hoechst (nuclear DNA, purple), CD61 (integrin beta 3, red) and GFP-KRAS^G12D^ (green) in both undifferentiated and differentiated MEG-01 cells transduced with the EV or the GFP-KRAS^G12D^ vector (Figure 1C,D). The platelet surface marker CD61 was consistently localized at the cell periphery in both undifferentiated and differentiated MEG-01 EV and GFP-KRAS^G12D^ cells. In contrast, the GFP-KRAS^G12D^ protein was exclusively detected in the MEG-01 GFP-KRAS^G12D^ cell line and was distributed throughout the intracellular region and the perimembrane area (Figure 1C,D, bottom panels). The extended morphology and presence of cellular protrusions in both MEG-01 EV and GFP-KRAS^G12D^ cells further indicated their differentiation status (Figure 1D).

Quantitative assessment of mean fluorescence intensity (MFI) demonstrated a significant increase in CD61 expression upon differentiation in both EV and GFP-KRAS^G12D^ cells. In particular, the MFI of CD61 in differentiated GFP-KRAS^G12D^ cells was 5-fold higher than in undifferentiated cells (*p* < 0.001), reflecting the progression of megakaryocytic maturation. Notably, GFP-KRAS^G12D^ expression was observed exclusively in the MEG-01 GFP-KRAS^G12D^ cell line. While this expression persisted throughout the differentiation process, it showed a decline in the differentiated cells (Figure 1E).

PLPs produced from both engineered differentiated cell lines retained the CD61 surface marker. Notably, PLPs derived from differentiated MEG-01 GFP-KRAS^G12D^ cells exhibited GFP-KRAS^G12D^ expression, suggesting successful loading of the fusion protein into the PLPs. They also displayed size and morphology consistent with typical platelets, as illustrated in Figure 1F. These results confirm that the engineered MEG-01 cells successfully produce PLPs harboring the fusion protein while retaining key characteristics of native platelets.

### 2.2. Characterization of GFP-KRAS^G12D^-Loaded PLPs

**Figure 2:**
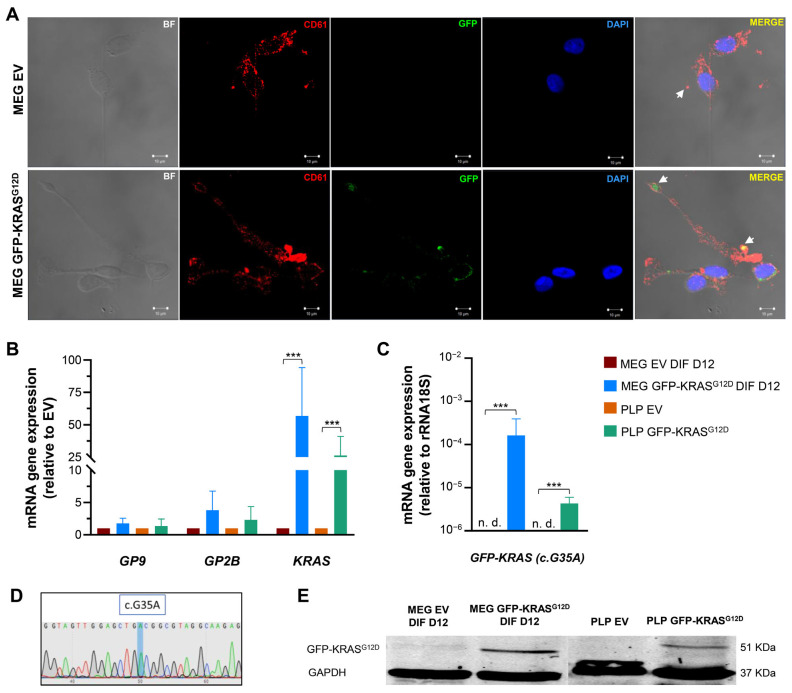
Production of PLP charged with GFP-KRAS^G12D^ from differentiated MEG-01 cell line. (**A**) Immunocytofluorescence of differentiated MEG-01 EV (top) and GFP-KRAS^G12D^ (bottom). Channels indicate the localization and expression levels for CD61 (red), GFP-KRAS^G12D^ (green) and DAPI (nucleic DNA, blue); the last channel corresponds to the merged channels. The arrows indicate the proplatelet formation. Scale bar = 10 μm. (**B**) qRT-PCR analysis showing the expression of *GP9*, *GP2B* and *KRAS* genes in differentiated MEG-01 EV and GFP-KRAS^G12D^ and PLP produced at day 12. (**C**) qRT-PCR analysis showing the expression of the fusion gene in differentiated MEG-01 EV and GFP-KRAS^G12D^ and PLP produced at day 12. (**D**) Characterization of fusion gene cDNA coding sequence (mutation corresponds to c.G35A). (**E**) Western blot analysis detecting the GFP-KRAS^G12D^ fusion protein in both MEG-01 differentiated and PLP. GAPDH is used as a loading control. Molecular weights: GFP-KRAS^G12D^ (51 kDa) and GAPDH (37 kDa). Data in plots represent mean ± SD for three independent experiments. Statistics were assessed with two-tailed unpaired Student’s *t*-test (statistically significant differences: *** *p* < 0.001).

Immunocytofluorescence analysis of differentiated MEG-01 cells revealed proplatelet formation (Figure 2A, white arrows), a hallmark of platelet production, along with strong CD61 expression in both EV and GFP-KRAS^G12D^ differentiated cells. GFP-KRAS^G12D^ expression was exclusively detected in MEG-01 GFP-KRAS^G12D^ cells (Figure 2A). Interestingly, GFP-KRAS^G12D^ was also observed within the proplatelets (Figure 2A, white arrows).

To further characterize the molecular profile of the engineered cells and PLPs, we performed quantitative RT-PCR analysis. Platelet-specific genes (*GP9*, *GP2B*) and KRAS were expressed in both differentiated cells and PLPs (Figure 2B). Importantly, the GFP-KRAS^G12D^ fusion gene was specifically expressed in the transduced cells and PLPs, with insignificant expression in the EV controls (Figure 2C). Sequence analysis confirmed the presence of the G12D mutation (c.G35A) in the fusion construct at the mRNA level (Figure 2D).

Western blot analysis confirmed the expression of the GFP-KRAS^G12D^ fusion protein expression in both differentiated cells and PLPs GFP-KRAS^G12D^. A distinct band corresponding to the expected molecular weight of 51 KDa was observed in the GFP-KRAS^G12D^ samples, while this band was absent in the EV controls, confirming the successful production and incorporation of the fusion protein into PLPs (Figure 2E). GAPDH (37 KDa) was used as the endogenous control. We confirmed that GFP-KRAS^G12D^ expression remained stable in the PLPs, making them suitable for co-culture with other cell types to study the transference and impact of this oncogenic protein.

### 2.3. Transfer of GFP-KRAS^G12D^ from Loaded PLPs to H1975 Tumor Cells

**Figure 3:**
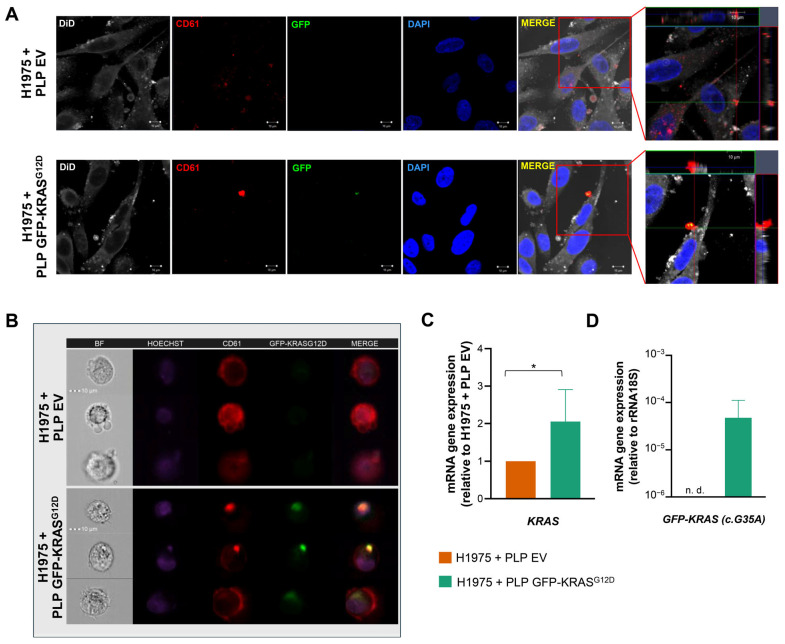
PLP fusion and GFP-KRAS^G12D^ transfer to H1975 tumor cells. (**A**) Immunocytofluorescence of H1975 cells co-cultured with PLP EV (top) and GFP-KRAS^G12D^ (bottom). Channels indicate the localization and expression levels for DiD (gray), CD61 (red), GFP-KRAS^G12D^ (green), DAPI (nucleic DNA, blue) and merge channel. The last image corresponds to *ortho* Z-stack slide. Scale bar = 10 μm. (**B**) Representative ImageStream images of co-culture of H1975 cells with PLP EV (top) and GFP-KRAS^G12D^ (bottom). Channels indicate the localization and expression levels for Hoechst (nucleic DNA, purple), CD61 (red) and GFP-KRAS^G12D^ (green); the last channel corresponds to the merged channels, except for the brightfield (BF). (**C**) qRT-PCR analysis showing the expression of *KRAS* gene in co-culture of H1975 cells with PLP EV and GFP-KRAS^G12D^. (**D**) qRT-PCR analysis showing the expression of the fusion gene in co-culture of H1975 cells with PLP EV and GFP-KRAS^G12D^. Data in plots represent the mean ± SD for three independent experiments. Statistics were assessed with two-tailed unpaired Student’s *t*-test (statistically significant differences: * *p* < 0.05).

To evaluate the potential of PLPs to transfer GFP-KRAS^G12D^ biomolecules in the lung tumor context, we conducted co-culture experiments with H1975 lung adenocarcinoma cells. Immunocytofluorescence analysis confirmed the presence of GFP-KRAS^G12D^ within H1975 cells after co-culture with loaded PLPs, indicating successful transfer of the fusion protein (Figure 3A). Z-Stack analysis further revealed that PLPs were in the same focal plane as the cells, suggesting that PLPs fused with or were internalized by H1975, facilitating the transfer of the GFP-KRAS^G12D^ fusion protein (Figure 3A, right panel).

This observation was corroborated by ImageStream analysis, which demonstrated co-localization of GFP-KRAS^G12D^ and CD61 signals within H1975 cells following co-culture with GFP-KRAS^G12D^-loaded PLPs (Figure 3B). In contrast, when H1975 cells were co-cultured with EV PLPs, only the CD61 signal was detected, confirming that GFP-KRAS^G12D^ transfer was specific to PLPs loaded with the fusion protein.

RT-qPCR analysis revealed a significant increase in the expression of both total KRAS (*p* < 0.05) and GFP-KRAS^G12D^ fusion gene (*p* < 0.001) expression in H1975 cells following co-culture with GFP-KRAS^G12D^ PLPs, compared to co-culture with EV PLPs (Figure 3C,D). These results provide compelling evidence for the successful transfer of genetic material from PLPs to tumor cells.

### 2.4. Functional Impact of GFP-KRAS^G12D^ Transfer on H1975 Tumor Cell Proliferation

**Figure 4:**
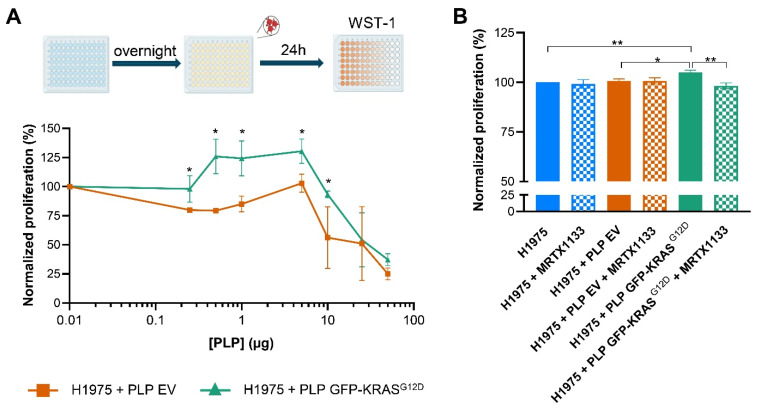
Evaluation of the effect in proliferation of PLP GFP-KRAS^G12D^ in H1975 tumor cells. (**A**) Proliferation assay of H1975 cells co-culture with different concentrations of PLP EV and PLP GFP-KRAS^G12D^. (**B**) Proliferation assay of H1975 cells co-culture with PLP EV and PLP GFP-KRAS^G12D^ and treated with MRTX1133 specific KRAS^G12D^ inhibitor. Data in plots represent the mean ± SEM for three independent experiments. Statistics were assessed with two-tailed unpaired Student’s *t*-test (statistically significant differences: * *p* < 0.05, ** *p* < 0.01).

To investigate the biological significance of the observed PLP fusion and the subsequent effect of the oncoprotein, we evaluated the proliferative capacity of H1975 cells after co-culture with PLPs. Proliferation assays revealed that a 24hour co-culture with GFP-KRAS^G12D^-loaded PLPs significantly enhanced H1975 cell proliferation compared to the EV PLP co-culture, particularly at lower PLP concentrations of 0.25 to 5 µg of PLP per well (*p* < 0.05) (Figure 4A). These results indicate that the transferred oncogenic KRAS^G12D^ retains its pro-proliferative function within recipient tumor cells. However, at higher PLP concentrations (10 to 50 µg of PLP per well), cell proliferation decreased, suggesting potential toxicity at elevated PLP levels (Figure 4A).

The co-culture condition using 5 µg of PLPs per well yielded the best results. Both EV and GFP-KRAS^G12D^-loaded PLPs promoted higher proliferation compared to the control without PLPs, with significantly greater proliferation observed in cells exposed to GFP-KRAS^G12D^-loaded PLPs than those exposed to EV PLPs (Figure 4A). This condition was selected for further experiments.

To confirm the results and corroborate the oncoprotein function in the co-culture, we used the MRTX-1133 inhibitor to specifically target KRAS^G12D^. As anticipated, the co-culture of H11975 cells with PLP GFP-KRAS^G12D^ exhibited increased proliferation compared to both the control and co-culture with PLP EV (Figure 4B). Furthermore, H11975 cells co-cultured with PLP GFP-KRAS^G12D^ in the absence of the inhibitor showed significantly greater proliferation than those treated with MRTX-1133 (Figure 4B), highlighting the critical function of KRAS^G12D^ in growth enhancement. Notably, no significant differences in proliferation were observed among the other experimental groups.

## 3. Discussion

Our study aimed to investigate the role of platelets in transferring oncogenic KRAS to tumor cells and its subsequent impact on NSCLC progression and resistance to therapy. For that purpose, we engineered the MEG-01 cell line to express the GFP-KRAS^G12D^ fusion protein and examined the production of PLPs containing this oncogene. Our results confirm that MEG-01 cells were successfully modified to express exogenous proteins, consistent with findings from other studies [29,30].

Recent studies demonstrated the role of platelets as carriers of tumor-derived proteins and nucleic acids [6,9,14]. This biomolecules transfer can occur within the tumor microenvironment, where platelets are in circulation, or during metastasis, when platelets assist circulating tumor cells in their dissemination [13,31,32]. The stable expression of GFP-KRAS^G12D^ during the differentiation process, along with its efficient incorporation into PLPs, highlights the potential of these particles as vehicles for transferring oncogenic material to tumor cells [9,33,34].

In our study, we demonstrated that the GFP-KRAS^G12D^-loaded PLPs were able to transfer GFP-KRAS^G12D^ to H1975 tumor cells. The successful transport of the protein between PLPs and NSCLC was confirmed by immunofluorescence and ImageStream analysis, which showed the presence of GFP-KRAS^G12D^ in H1975 cells after co-culture with GFP-KRAS^G12D^-loaded PLPs, similar to the platelet phagocytosis and protein transfer shown in Martins Castanheira et al. [12,24]). Additionally, qRT-PCR analysis showed a significant increase in both *KRAS* and *GFP-KRAS^G12D^* fusion gene expression in H1975 cells post-co-culture with GFP-KRAS^G12D^ PLPs, validating the transfer of mRNA alongside the protein. As other researchers defend [12,27,35,36], these findings strongly support the hypothesis that platelets can mediate the transfer of oncogenic material (both mRNA and protein) to tumor cells, potentially influencing tumor behavior and progression. In this regard, we offer more information concerning the multifaceted role of platelets in cancer progression [27,32,37], and we extend their function beyond their conventional role in hemostasis [1,2,38].

To assess the biological significance of co-culture with platelets and the oncogene transfer, we evaluated the proliferative capacity of H1975 cells following co-culture with PLPs. Proliferation surveys revealed a significant increase in H1975 cell growth after co-culture with determined concentrations of GFP-KRAS^G12D^ and EV PLPs. We obtained the best result with 5 µg of PLPs.

However, it is noteworthy that at high PLP concentrations, the co-culture exhibited decreased proliferation. We observed that elevated PLP levels induce cytotoxicity, aligning with recent studies showing that PLPs derived from MEG-01 exert antitumor effects on prostate cancer cells, reducing invasiveness and promoting apoptosis [39]. Intriguingly, GFP-KRAS^G12D^ appears to mitigate PLP-induced cytotoxicity. At 10 μg of PLPs, the GFP-KRAS^G12D^ condition showed no increase in co-culture proliferation, whereas the PLP-EV condition exhibited markedly higher cytotoxicity.

Our results suggest that the oncogenic GFP-KRAS^G12D^ transfer at determined PLP concentrations maintains its pro-proliferative function within recipient tumor cells, as previously shown [24,34]. Furthermore, the enhanced proliferation aligns with the well-established role of mutant *KRAS* in promoting tumor growth and progression [19]. Our findings extend this concept, demonstrating that platelet-mediated transfer of oncogenic KRAS can confer growth advantage to recipient tumor cells. These results were confirmed using the KRAS^G12D^-specific inhibitor MRTX-1133. In the presence of the inhibitor, cellular proliferation rates were analogous to those observed in control cells and cells co-cultured with PLP-EV [40].

Our study also underscores the critical need to consider each cell component of the tumor microenvironment and its interactions with circulating cells, such as platelets, in understanding tumor progression [41,42]. Another level of complexity is involved in this research, as the implications of our results extend beyond *KRAS* and NSCLC, suggesting the platelet-mediated oncogene transfer could be a broader phenomenon, potentially relevant to other oncogenes and cancer types. Further studies will be needed to clarify this issue.

Our findings emphasize the potential role of platelet-mediated oncogene transfer in promoting both tumor progression and therapy resistance. Targeting platelet–tumor interactions [21,43,44] or disrupting the transfer of oncogenic material may represent promising approaches to improve treatment outcomes for patients with NSCLC.

Taken together, the results of our study provide important insights into the role of platelets in oncogene transfer and tumor progression but still raise several questions for future research. First, an interesting issue to fully understand is the exact mechanisms of how platelets, or PLPs in our model, transfer oncogenic material to tumor cells, whether through fusion, endocytosis or other processes. Future efforts should also assess the clinical relevance of this phenomenon by analyzing both tumor biopsies and circulating platelets from NSCLC patients and tracking oncogenic material during disease progression. That could allow the development of novel liquid biopsy techniques and offer new insights into resistance mechanisms.

## 4. Materials and Methods

### 4.1. Cell Culture

MEG-01 (RRID:CVCL_0425) and NCI-H1975 (H1975) (RRID:CVCL_1511) lung adenocarcinoma cell lines were obtained from ATCC and cultured in RPMI-1640 medium (Biowest, Nuaille, France) supplemented with 10% fetal bovine serum (FBS, Biowest, Nuaille, France) and 1X penicillin/streptomycin (PS) (Sigma-Aldrich, Merck, St. Louis, MO, USA). H1975 cells were passed every 5–6 days at a seeding ratio of 1:5. MEG-01 cells were grown in suspension and passed weekly at a 1:5 ratio.

HEK293T (RRID:CVCL_0063) cells were cultured in DMEM-high glucose medium (Biowest) supplemented with 10% FBS and 1X PS and passed every 3–4 days at a 1:5 to 1:8 ratio using 0.75X TrypLE Express (Thermo Fisher Scientific, Waltham, MA, USA) for 5 min.

All cell lines were maintained at 37 °C in a humidified atmosphere with 5% CO_2_ and routinely tested for mycoplasma contamination using the Venor^®^GeM qEP (Minerva Biolabs, Dublin, Ireland). Cell line authentication was performed using AmpFLSTR Identifiler^®^ Plus (Thermo Fisher Scientific), confirming the cells were mycoplasma-free and STR-validated.

### 4.2. Lentiviral Vector Constructs

The pRRL-EF1a-PGK-NEO vector, kindly provided by Prof. Trono (EPFL, Lausanne, Switzerland), was used as the control empty vector (EV). The GFP-KRAS^G12D^ cDNA, originally obtained in a pBABE vector from Addgene (Watertown, MA, USA), was sub-cloned into the pRRL-EF1a-PGK-NEO vector using standard molecular techniques.

*E. coli* DH5α were transformed with the final constructs, and plasmid DNA was extracted using NucleoSpin Plasmid kit (Macherey-Nagel, Düren, Germany). Sequencing of the constructs was performed by StabVida laboratories (Caparica, Portugal). High-concentration plasmids were obtained through maxiprep from transformed *E. coli* DH5α cells.

### 4.3. Lentiviral Vectors Production and Cell Transduction

Lentiviral vectors (LVs) were generated by transfecting HEK293T cells with pRRL-EF1a-PGK-NEO (EV) or pRRL-EF1a-GFP-KRASG12D-NEO (GFP-KRAS^G12D^), along with psPax2 (packaging vector) and VSV.G (envelope vector) from Addgene, using the standard calcium phosphate transfection methods. Supernatants were collected 24 and 48 h post-transfection.

Viral titers were determined by serial dilutions of the concentrated LVs on 293T. GFP-positive cells were quantified by flow cytometry 3–5 days post-transduction.

Viral supernatants were used for transduction, supplemented with Polybrene Reagent (8 mg/mL, Sigma-Aldrich, Merck). Cells were transduced overnight, and the culture medium was replaced the following morning. Two days post-transduction, cells were selected with G418 (100 mg/mL; Invitrogen, Waltham, MA, USA) for 5 days. GFP-positive cells were then sorted using a FACS Aria Fusion (BD Biosciences, Franklin Lakes, NJ, USA).

### 4.4. MEG-01 Cell Line Differentiation and PLP Production

The MEG-01 cell line was differentiated with 2 mM valproic acid (VPA) over 12 days, a modified protocol from Schweinfurth et al., 2010 [45]. On day 0, 1.4 × 10^6^ cells were seeded in a T75 flask containing 14 mL of RPMI medium supplemented with FBS, PS and 2 mM VPA. The medium was refreshed on days 4 and 8. PLPs and differentiated cells were collected on day 12. To harvest PLPs, the culture supernatant was collected into a 15 mL Falcon tube and centrifuged at 180× *g* for 5 min to remove cellular debris. Subsequently, the resulting supernatant was filtered through a 5 µM P Mm filter (Merck) and transferred to a low-adhesion 15 mL Falcon tube. This was followed by centrifugation at 1200× *g* for 10 min. The harvested pellet was washed with PBS, pooled and centrifuged again at 1200× *g* for 10 min. For co-culture experiments, the final pellet was resuspended in RPMI medium supplemented with FBS and PS.

To collect the differentiated cells, the T75 flask was washed with PBS, and 0.75X TrypLE was added and incubated for 5 min. Afterward, 1X PBS was added, and the cells were gently detached using a cell scraper. The cell suspension was transferred to a 15 mL Falcon tube and centrifuged at 250× *g* for 5 min to collect the cells.

### 4.5. MEG-01 Cell Line Differentiation for Immunofluorescence and Confocal Analysis

Cells were plated at low density in an 8-well format (Nalge Nunc International, Naperville, IL, USA), adjusting the density to a final concentration of 1 × 10^5^ cells per well in 200 μL of medium. Differentiation followed the previously described protocol. On day 12, cells were washed with PBS, fixed in 4% paraformaldehyde (Sigma-Aldrich) in PBS and permeabilized with 0.1% Tween (Sigma-Aldrich) for 5 min at room temperature. Blocking was performed with 5% BSA (Sigma-Aldrich) (*w*/*v*) in PBS for 30 min with washes carried out in PBS containing 0.1% BSA. Immunostaining was done overnight at 4 °C using anti- Hu CD61 (ref. 14-0619-82, Invitrogen) and anti-GFP (ref. 2955S, Abcam, Cambridge, UK) antibodies at final concentrations of 1:200 diluted in 3% BSA/PBS. After that, the samples were washed with PBS containing 0.1% BSA and incubated with secondary antibodies Alexa Fluor 555 Donkey anti-mouse (A31570, Invitrogen) and Alexa Fluor 488 Donkey anti-rabbit (A21206, Invitrogen) antibodies diluted in 3% BSA/PBS for 1 h at room temperature. The samples were washed with PBS containing 0.1% BSA. Cells were mounted and counterstained with DAPI using VECTASHIELD^®^ Antifade Mounting Media (Vector Laboratories, Burlingame, CA, USA). Immunofluorescence images were captured using an Axio Observer Z1 inverted fluorescence microscope (Carl Zeiss, Jena, Germany) and processed using ZEN software (version 6.0) (Carl Zeiss).

### 4.6. Flow Cytometry and ImageStream Analysis of MEG-01 Cell Line Differentiation

Cells were collected, washed once with PBS and centrifuged at 150× *g* for 5 min. They were resuspended to a final concentration of 1 × 10^6^ cells/mL in PBS, and 100 µL of cells suspension was incubated with anti-human CD61-AF647 antibody (ref. 336408, VI-P12D, Invitrogen) at a 1:200 final concentration for 30 min at room temperature. Following incubation, cells were washed with PBS, centrifuged and resuspended in FACS buffer (PBS supplemented with 5% FBS and 2 mM EDTA).

Labeled cells were analyzed using either a FACS Canto II flow cytometer or an ImageStream Mark II imaging flow cytometer (Cytek Amnis, Seatle, WA, USA). Data were processed using Cytobank (https://community.cytobank.org/cytobank/login) and IDEAS software (version 6.4, Cytek Amnis), respectively.

Both cells and PLPs were stained as described in the flow cytometry analysis section. After staining, cells were fixed with 4% paraformaldehyde in PBS for 12 min and permeabilized with 0.1%Triton X-100 (Sigma-Aldrich) in PBS for 20 min. Then, cells were incubated with 1 μM Hoechst (Merck) for 3 min, washed and resuspended in FACS buffer. Platelets were similarly stained, washed and resuspended in PBS with 2 mM EDTA. Data were acquired in an ImageStream Mark II imaging flow cytometer and analyzed with IDEAS software.

### 4.7. RNA Extraction, RT-PCR, qPCR and Sanger Sequencing

Total RNA was extracted using the NucleoSpin^®^ RNA kit (Macherey-Nagel) following manufacturer’s instructions. cDNA was synthesized from the extracted RNA using the Transcriptor First Strand cDNA Synthesis Kit (Roche; Basel, Switzerland) following the manufacturer’s protocol. Gene expression was assessed by qPCR using Brilliant III Ultra-Fast SYBR^®^ Green QPCR Master Mix (Agilent Technologies; Santa Clara, CA, USA) on a 7900HT Fast Real-Time PCR System (Thermo Fisher Scientific). Data analysis was performed using the 2^−ΔΔCT^ method, with GAPDH as the normalization control. The GFP-KRAS^G12D^ cDNA product obtained from the RT-qPCR was purified and sequenced by StabVida laboratories. The qPCR and sequencing primers are listed in Table S1 and Table S2, respectively.

### 4.8. Western Blot Analysis

For total protein extraction, MEG-01 cells were lysed in RIPA buffer (Sigma-Aldrich) containing a protease inhibitor cocktail (Roche) and phosphatase inhibitor cocktails 2 and 3 (Sigma-Aldrich). Cell lysates were separated by molecular weight using SDS-polyacrylamide gels and transferred to cellulose membranes. Proteins were detected using the Odyssey Infrared Imaging System (Li-cor Biosciences; Lincoln, NE, USA). GFP-KRAS^G12D^ was detected with anti-GFP antibody (ref. 2955S, Abcam), while α-GAPDH (ref. 60004-1-Ig, Proteintech, Rosemont, IL, USA) was used as a loading control. Western blotting was carried out using standard procedures. All the results shown correspond to the same membrane.

### 4.9. Immunofluorescence and ImageStream of the Co-Culture

Cells were plated at a density of 1 × 10^5^ cells/well and 200 μL of medium in an 8-well format. H1975 cells were co-cultured with either EV or GFP-KRAS^G12D^-loaded PLPs. After 24 h, the cells were washed with PBS, fixed in 4% paraformaldehyde (Sigma-Aldrich), permeabilized with 0.2% Triton X-100, blocked with 3% of BSA (Sigma-Aldrich) and stained as previously described in the immunofluorescence and confocal section, with the exception that Celular Vibrant DiD (V22887, Invitrogen), which was added at 10 µM for staining tumor cells membranes. Immunofluorescence images were acquired using an Axio Observer Z1 inverted fluorescence microscope (Carl Zeiss) and processed with ZEN software.

For ImageStream analysis of the co-culture, H1975 cells were seeded at a density of 2.5 × 10^4^ cells/well in 24-well plates. Then, cells were co-cultured with 25 µg of either EV or GFP-KRAS^G12D^-loaded PLPs in 1 mL of medium. After 24 h, the co-cultures were harvested, washed with PBS and centrifuged at 250× *g* for 5 min. The pellet was resuspended and fixed with 4% paraformaldehyde in PBS for 10 min and permeabilized with 0.1%Triton X-100 (Sigma-Aldrich) in PBS for 5 min. Then, cells were resuspended in PBS to a final concentration of 1 × 10^6^ cells/mL. Aliquots of 100 µL cell suspension were incubated with anti-human CD61-AF647 antibody (Invitrogen) at a 1:200 dilution for 30 min at room temperature. After that, cells were washed twice with PBS, centrifuging at 250× *g*. Then, cell nuclei were stained with 1 μM Hoechst (Merck Life Sciences) for 3 min, washed and resuspended in FACS buffer. Data were acquired in an ImageStream Mark II imaging flow cytometer (Cytek Amnis) and analyzed with IDEAS software (Cytek Amnis).

### 4.10. Functional Assay: Determination of Viability and Proliferation of the Co-Culture with Different Amounts of PLPs

H1975 cells were seeded at 5 × 10^4^ cells per well in 96-well plates. After 24 h, different concentrations of PLPs (0, 0.25, 0.5, 1, 5, 10, 25 and 50 µg/well) were added. The impact of co-culturing tumor cells with varying amounts of EV or GFP-KRAS^G12D^-loaded PLPs was evaluated 24 h post-co-culture using the WST-1 reagent (Roche) according to the manufacturer’s instructions. Absorbance at 450 nm was measured using a Tecan Infinite 200 Pro microplate reader (Tecan Trader AG, Männedorf, Switzerland). Cell viability was expressed as normalized absorbance relative to control cells.

### 4.11. Functional Assay: Determination of Viability of the Co-Culture Treated with MRTX-1133-Specific KRAS^G12D^ Inhibitor

H1975 cells were seeded at a density of 5 × 10^4^ cells/well in 96-well plates and incubated for 24 h. Subsequently, PLPs, either untreated or pretreated with 1 µM MRTX-1133 inhibitor (generously provided by Dr. Chiara Ambrogio), were added at a concentration of 5 µg/well. Cell viability was assessed using the WST-1 reagent (Roche), following the protocol outlined in the previous section. Viability was quantified as normalized absorbance relative to untreated control cells.

### 4.12. Statistics

All statistical analyses and graph plotting were conducted using GraphPad Prism (version 8.0 for Windows, GraphPad Software). Expression and functional experiments were performed in both biological and technical triplicates. Data were represented as mean ± SD or mean ± SEM. For comparisons between two different groups, a *t*-test was applied to assess statistical significance. A *p*-value < 0.05 was considered statistically significant.

## 5. Conclusions

Our study successfully engineered MEG-01 cells to produce PLPs carrying oncogenic KRAS, demonstrating the efficient transfer of this oncogene to tumor cells and its significant impact on enhanced proliferation and drug resistance. These findings provide novel insights into the mechanisms of tumor progression mediated by platelet–tumor cell interactions. By elucidating the role of platelet-mediated oncogene transfer, this research opens avenues for the development of new therapeutic strategies and personalized medicine approaches for NSCLC patients.

## Data Availability

The authors confirm that all data supporting the results included in this manuscript are available in the article document or in its accompanying supplemental materials.

## Supplementary Materials

The following supporting information can be downloaded at https://www.mdpi.com/article/10.3390/ijms26073264/s1.
