# Supplementary material for: Oncogenic KRASG12D Transfer from Platelet-like Particles Enhances Proliferation and Survival in Non-Small Cell Lung Cancer Cells
Source: Int J Mol Sci. 2025 Apr 1;26(7):3264. doi: 10.3390/ijms26073264 (PMC11990068; doi:10.3390/ijms26073264)
Supplement: Supplementary file 1 [file ijms-26-03264-s001.zip › ijms-3477161-supplementary.pdf]

Oncogenic KRAS^{G12D} Transfer from Platelet-like Particles Enhances Proliferation and Survival in Non-Small Cell Lung Cancer Cells

Supplementary Data

Jorge Ceron-Hernandez [1,2,3], Gonzalo Martinez-Navajas [1,3], Jose Manuel Sanchez-Manas [1,3], María Pilar Molina [2], Jiajun Xie [1,3], Inés Aznar-Peralta [2], Abel Garcia-Diaz [2], Sonia Perales [1,3,4], Carolina Torres [1,4,5], Maria J. Serrano [2,4,6,*] and Pedro J. Real[1,2,3,*]

1 Gene Regulation, Stem Cells and Development Group, GENyO, Pfizer-University of Granada-Andalusian Regional Government Centre for Genomics and Oncological Research, Avenida de la Ilustración 114, 18016 Granada, Spain; jorge.ceron@genyo.es (J.C.-H.); gm24@sanger.ac.uk (G.M.-N.); jose.sanchez@genyo.es (J.M.S.-M.); jiajun.xie@genyo.es (J.X.); sopero@ugr.es (S.P.); ctp@ugr.es (C.T.)

2 Liquid Biopsies and Cancer Interception Group, PTS, Granada GENyO, Pfizer-University of Granada-Andalusian Regional Government Centre for Genomics and Oncological Research, Avenida de la Ilustración 114, 18016 Granada, Spain; maria.molina@genyo.es (M.P.M.-V.); ines.aznar@genyo.es (I.A.-P.); abel.garcia@genyo.es (A.G.-D.)

3 Department of Biochemistry and Molecular Biology I, Faculty of Science, University of Granada, Avenida Fuentenueva s/n, 18071 Granada, Spain

4 Instituto de Investigación Biosanitaria ibs.GRANADA, 18012 Granada, Spain

5 Department of Biochemistry and Molecular Biology III and Immunology, Faculty of Medicine, University of Granada, Avenida de la Investigación 11, 18016 Granada, Spain

6 Molecular Pathology Lab. Intercenter Anatomical Pathology Unit, San Cecilio and Virgen de las Nieves University Hospitals, 18016 Granada, Spain

* Correspondence: mjose.serrano@genyo.es (M.J.S.); pedro.real@genyo.es (P.J.R.)

Table S1. Primers used for RT-qPCR.

Name	RT-qPCR primers (5'-3')	Amplified cDNA
GP9E1-2_FW	ACAGGAGCACCTGACCAAAG	*GP9*
GP9E1-2 _RV	GGGTCTCAGCCTTCTCCTCT	*GP9*
GP2B_FW	TGAGCCGCATTTACGTGGAAA	*GP2B*
GP2B_RV	CTTCACAGTAACGCTTGTCCC	*GP2B*
KRAS WT_FW	GTACATGAGGACTGGGGAGGG	*KRAS*
KRAS WT_RV	CGTGAGCCTGTTTTGTGTC	*KRAS*
EGFP-KRASG12D_FW	CACGACGGCAACTACAAGACC	*GFP-KRAS (c.G35A)*
EGFP-KRASG12D_RV	CTTGAAGTCGATGCCCTTCAGC	*GFP-KRAS (c.G35A)*

Table S2. Primers used for sanger sequencing.

Name	Sanger sequencing primers (5'-3')
HA-KRAS_FW	TCACTCCTTCTCTAGGCGC
